# Paleoclimate-conditioning reveals a North Africa land–atmosphere tipping point

**DOI:** 10.1073/pnas.2108783118

**Published:** 2021-11-01

**Authors:** Peter O. Hopcroft, Paul J. Valdes

**Affiliations:** ^a^School of Geography, Earth & Environmental Sciences, University of Birmingham, Birmingham B15 2TT, United Kingdom;; ^b^Bristol Research Initiative for the Dynamic Global Environment, School of Geographical Sciences, University of Bristol, Bristol BS8 1SS, United Kingdom;; ^c^Cabot Institute, University of Bristol, Bristol BS8 1SS, United Kingdom

**Keywords:** tipping point, abrupt climate change, climate model

## Abstract

While paleoclimate records show that the Earth System is characterized by several different tipping points, their representation within Earth System models (ESMs) remains poorly constrained. This is because historical observations do not encompass variations large enough to provoke such regime changes, and paleoclimate conditions are rarely used to help develop and tune ESMs, which potentially ignores a rich source of information on abrupt climate change. A critical example is the early to mid-Holocene “greening” and subsequent rapid desertification of the Sahara, which most ESMs fail to reproduce, casting doubt on the representation of land–atmosphere coupling and monsoon dynamics. Here, we show that this greening and abrupt termination can be successfully simulated with one ESM after optimizing uncertain model components using both present-day observations and crucially mid-Holocene (6,000 y before present) reconstructions. The optimized model displays abrupt threshold behavior, which shows excellent agreement with long paleoclimate records that were not used in the original optimization. These results suggest that in order to realistically capture climate-system thresholds, ESMs first need to be conditioned with appropriate paleoclimate information.

## Significance

Understanding of climate “tipping points” is extremely limited. Their representation within Earth System models (ESMs) is completely unconstrained because of a lack of any actual occurrences of these events in recent times. As an example, most ESMs fail to simulate the rapid desertification of the Sahara during the Holocene. Here, we overcome this by tuning uncertain aspects of one ESM using the climate of 6,000 y ago. This approach reveals a hitherto-absent tipping point within this ESM that produces excellent agreement between the modeled and observed timing of abrupt change in North Africa. This demonstrates how paleoclimate information can be used to radically improve the representation of abrupt changes in the ESMs that are employed for future projections.

The stability of Earth’s climate is critical for understanding past and future environments (1–5). The later part of the Holocene epoch was stable, perhaps providing the unique environmental background for agriculture and civilization. In contrast, paleoclimate records reveal how the stability of the Holocene was unusual. The climate system is capable of rapid transitions that greatly outpace the rate of change of driving factors. Under the influence of increasing greenhouse-gas radiative forcing, the potential for abrupt climate change is likely to increase in the future (4–6). However, understanding of the probability and potential occurrence of tipping elements is extremely limited (4, 6). A major reason for this is that Earth System models (ESMs) used in future projections are not evaluated against any actual abrupt climate-change events (7). Instead, models are tuned against the spatial and seasonal distribution of key variables, such as radiation balance, temperature, and precipitation (8). This means that poorly constrained ESMs have to be used to quantify the risk of abrupt thresholds (5).

A prominent example of an abrupt climate change occurred in North Africa during the mid-Holocene. Multiple independent lines of evidence support a significant greening of the Sahara during the early to mid-Holocene. Fossil pollen records show a widespread northward expansion of shrub and savannah biomes (9–12). Sedimentary records indicate the reactivation of aquifers and deepening and expansion of lakes (13). Ocean cores show a 60 to 80% reduction in mineral dust flux reaching sites as far as the Caribbean (14, 15), and leaf waxes from sediment cores near West Africa have a negative hydrogen isotope excursion consistent with enhanced rainfall (16).

The Holocene African humid period (AHP) came to an end around 4,000 y (4 ka) to 6 ka before present (BP) (17, 18). Early evidence suggested that it terminated relatively rapidly compared with the gradual millennial-scale decline in insolation (19), but a marine sediment dust record from the region (14) provided the first compelling evidence for centennial or even decadal scale transition out of the AHP. Reconstructions from a wider range of proxies and over a larger area (16, 17) display an abrupt transition, but in regions further to the south or east, the hydrological response is much more gradual (20, 21).

Charney et al. (22) first suggested a positive feedback mechanism between vegetation and the monsoon in North Africa. A rapid termination of the AHP is consistent with this process because small changes in external forcings can be amplified, accelerating the pace of the system response. Theoretically, this could be linked to bistability in the system and the possibility of abrupt transitions between states (23, 24). A major barrier to testing this theory in more detail is the underestimation of mid-Holocene rainfall in North Africa by nearly all general circulation models (GCMs) (25, 26) and ESMs (27). As a result, the dynamics of this system cannot be evaluated with the detailed three-dimensional models used to project future change (7).

A relatively abrupt reduction in vegetation cover and precipitation at around 5,500 y BP was simulated with the intermediate-complexity model CLIMBER-2 (2). Subsequent studies with other ESMs of intermediate complexity or low-resolution GCMs have shown either no abrupt change (28) or only regional impacts in the Eastern Sahara (29). The latter study appears to be in contradiction with subsequent reconstructions, which instead show gradual hydrological change in the Eastern region (20, 21). One higher-resolution ESM shows a realistic greening and desertification (30), but the transition is not as abrupt as some paleoclimate records suggest (14).

The reasons for the failure of ESMs to simulate the AHP remain unclear, and it is possible that new processes need to be considered (31–33). Some studies have argued that it is because of missing processes such as dust, but subsequent work has shown that this was due to overestimated shortwave absorption by dust particles in older observational datasets that are still employed in some ESMs (34, 35). Alternatively, existing models may already include adequate representations of the key processes, but these are not currently optimized or tuned appropriately, meaning that feedbacks are too strong or too weak, and emergent properties are biased. The latter would imply that paleoclimate is uniquely useful for narrowing suitable parameter ranges in ESMs/GCMs (36, 37).

Here, we analyze a suite of four transient simulations of the Holocene from 10 ka BP to present day using versions of HadCM3-M2.1 (the Hadley Centre Coupled Model Version 3 coupled to Version 2.1 of the Met Office Surface Exchange Scheme [MOSES]) (38–40). These configurations are based on the existing release model version (STD), but include paleoclimate-informed changes to atmospheric convection (+CONV) and dynamic vegetation (+VMS). +CONV increase the sensitivity of convection to summer insolation (37), and +VMS improves the simulation of vegetation cover in semiarid areas, which is required because many vegetation models have incorrect sensitivity (32). A fourth setup combines these (+CONV+VMS). Conceptually, this approach is unique because the parameters were optimized against both present-day and mid-Holocene climate reconstructions (37). The +CONV configuration of HadCM3 produces nearly twice the precipitation increase over North Africa relative to STD. Thus, we are potentially able to study the dynamics of the AHP termination in detail and to examine whether different parameterizations alter the fundamental properties of the model in this region.

## Results

### “Greening” and Abrupt Change

**Fig. 1.**
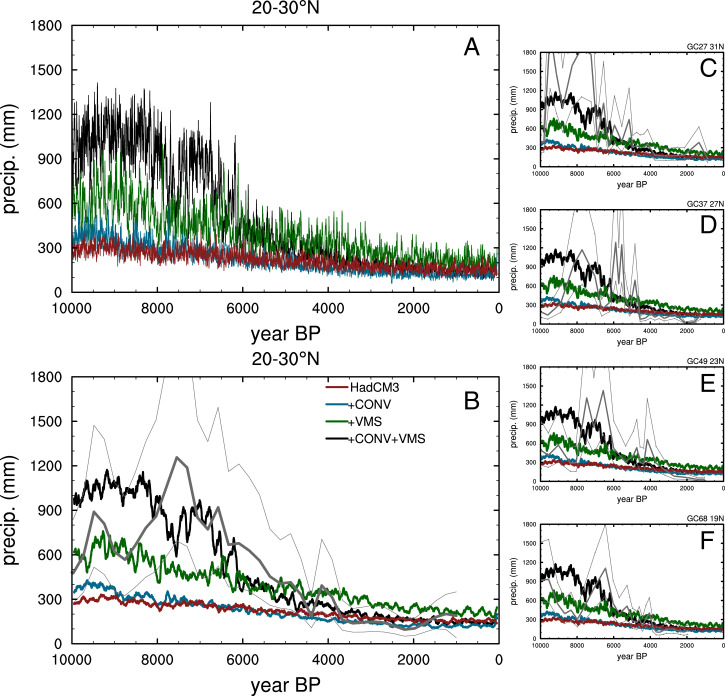
Simulated and reconstructed Holocene precipitation (precip.) in North Africa. (*A*) Annual mean simulated precipitation with four versions of HadCM3-M2.1. (*B*) The 100-y running mean in comparison with the four-core average reconstruction (16) shown in thick gray. (*C*–*F*) Comparison with individual cores. The uncertainty range for the reconstructed precipitation is shown by thin gray lines.

The four simulations all show a global mean warming of ∼1.2 K over the course of the Holocene (*SI Appendix*, Figs. S1 and S2). This is driven by reductions in ice volume and increasing greenhouse-gas forcing, mostly due to rising CO_2_, as shown in *SI Appendix*, Fig. S2. Total radiative forcing increases by around 1.2 Wm^– 2^ over the course of the simulation. The gradual reduction in Northern Hemisphere summer insolation forces a reduction in the strength of the Northern Hemisphere monsoons (Fig. 1 and *SI Appendix*, Fig. S3).

Over North Africa, the precipitation response is very different in the four models, as shown in Fig. 1 for the Western region from 20 to 30^∘^ N by 20^∘^ W to 5^∘^ E. In STD and +CONV, there is a very small enhancement of the West African monsoon in the early Holocene compared to the late Holocene. This only penetrates to around 15^∘^ N. STD is consistent with HadCM3-M2.1 simulations of 6 ka BP submitted to the Paleoclimate Modelling Intercomparison Project (PMIP) Phase II and is similar to other PMIP simulations (41). It shows a northward extension of precipitation over North Africa by only a few degrees, equivalent to approximately one model grid cell. The response is much stronger in +VMS, but +CONV+VMS shows the greatest precipitation increase and vegetation expansion (discussed below). A unique feature in +CONV+VMS are the abrupt reductions in precipitation at around 7.7, 6.6, and 6.1 ka BP. +VMS is substantially drier than +CONV+VMS until around 6 ka BP, and there is no evidence of this abrupt behavior in the VMS configuration. The VMS model is dryer in the early Holocene and wetter in the later Holocene, demonstrating that the convection changes in +CONV+VMS enhance the model’s sensitivity to the applied forcings, particularly the summer insolation decline, as shown also in *SI Appendix*, Fig. S3, and by the diagnosed land–atmosphere coupling coefficients shown in *SI Appendix*, Figs. S4 and S5.

The model results are compared with the individual and four core-mean rainfall reconstructions by Tierney et al. (16) in Fig. 1. The reconstruction depicts annual mean rainfall, but is compared here with the simulated June–July–August–September (JJAS) mean, since most of the precipitation in the model, around 70%, occurs in this season. However, the simulated annual mean rates are lower than reconstructed. Recent isotope modeling has suggested that the reconstructions may be too high (42). We judged that the temporal trends are robust, and these are useful for evaluating the timing and rapidity of the rainfall changes.

The average precipitation reconstruction from the four cores agrees remarkably well with the +CONV+VMS simulation, both in terms of the amplitude and the timing of reduction around 6.1 ka BP. Three of the individual records shows excellent agreement with the +CONV+VMS model in the timing of the abrupt collapse at around 6 ka BP. The remaining core (GC37 at 27 ^∘^ N) shows an out-of-phase response. It is unclear whether this could be due to chronological uncertainty, which is around 75 y for this part of record, with ^14^ C dates every 1 to 5 ka, or because of potential site-specific factors.

The reconstructions also show an abrupt precipitation reduction at or just after 8 ka BP, which approximately coincides with later phases of the 8.2 ka BP North Atlantic melt-water event, though the drying is significantly longer in duration (16). This centennial-scale drying event is supported by records from across the continent (12, 43–46). A very similar event occurs around 7.7 ka BP in the +CONV+VMS simulation independently of any melt-water forcing in the model. This appears to be a simulated “flickering” of the land–atmosphere system, most likely a precursor to the final collapse at 6.5 ka BP. The bistability diagnostic (discussed below) has already increased substantially by this point in the +CONV+VMS simulation, supporting this interpretation. The similarities between the reconstructed and simulated rainfall reduction at or after 8 ka BP therefore present a hitherto-unexplored explanation for the observed event—as an indicator or symptom of system instability. Tighter chronological controls in the paleoclimate records and/or further ensembles of simulations are needed for a definitive evaluation of this hypothesis.

**Fig. 2.**
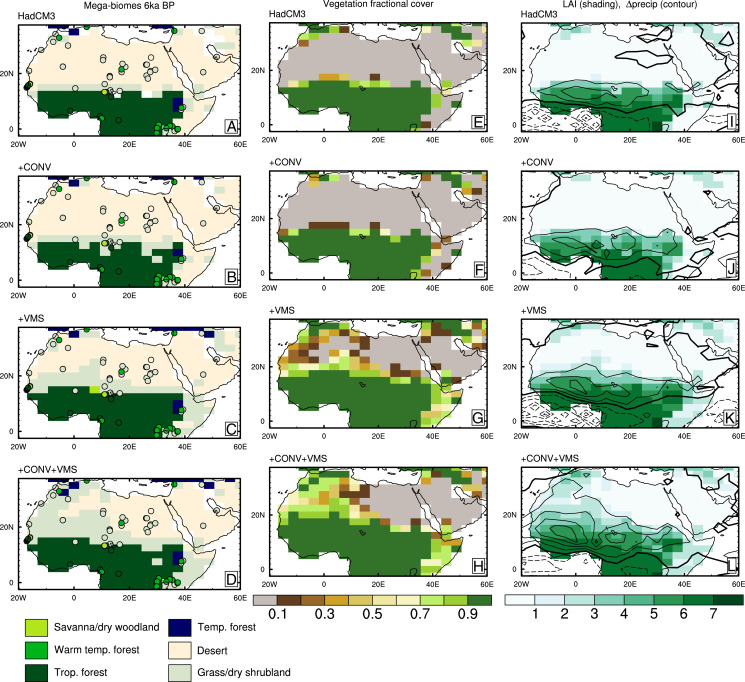
Mid-Holocene simulated and reconstructed (47) vegetation biomes (*A*–*D*), simulated vegetation coverage (*E*–*H*), and leaf area index (shading) and precipitation anomalies (contours; *I*–*L*). Temp., temperate; trop., tropical.

We also compare the simulated vegetation coverage with the mid-Holocene biome reconstruction (47) in Fig. 2. The simulated fractional coverage of plant functional types (PFTs) has been translated to mega-biomes following ref. 48. The comparison is performed for 6.5 ka BP because the vegetation cover and precipitation collapse just before 6.0 ka BP. The +CONV+VMS simulated biomes compare most favorably with the mid-Holocene pollen-based reconstruction, although there is still too little grass or temperate forest in the Central and Eastern Sahara around 20 to 30^∘^ E, up to around 24^∘^ N. Other reconstructions (49) also support this as the maximum northward extension of the monsoon precipitation during the Holocene. The other model configurations have too much bare soil across the whole continent between 20 and 35^∘^ N. Both the fractional coverage and leaf area index are correlated with the precipitation anomalies, suggesting a tight coupling, which we discuss further below. The lack of wetter conditions in the eastern region of the Sahara is consistent with earlier model simulations (50, 51) and is due to descending air masses in this region in Northern Hemisphere summer, consistent with a remote forcing by the Asian monsoon (52). The descent is not strengthened in the early and mid-Holocene, despite the stronger monsoon to the east, which suggests that there are competing influences operating in this region, including, for example, tropical plumes (30, 53).

**Fig. 3.**
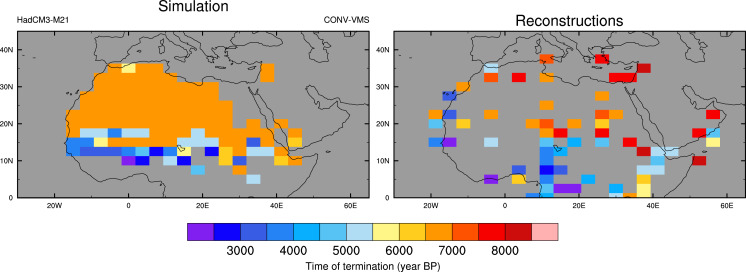
Simulated and observed time of termination of the Holocene AHP. The model results are based on the simulated bare soil fraction following ref. 30.

The timing of the end of the humid period is estimated from the modeled bare soil fraction following ref. 30 and is shown in Fig. 3. The +CONV+VMS model shows an earlier hydrological cycle decline in the north and a later transition further south, especially in the western equatorial region. This pattern is broadly supported by the reconstructed timing, which has been compiled from a suite of paleo-hydrological reconstructions (17, 30). Additional reconstructions in West Africa at around 10 to 20^∘^ N are required to resolve the sharp gradient simulated in the +CONV+VMS model version.

### Diagnostics of Bistability

**Fig. 4.**
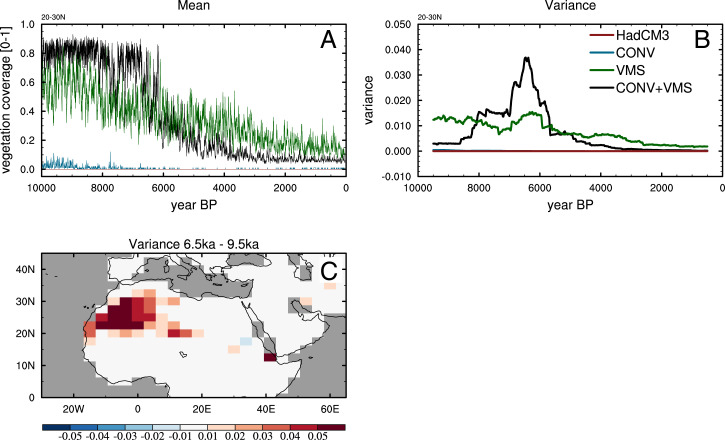
Simulated north African vegetation coverage. Mean (*A*) and running ±500-y variance (*B*) for northwest Africa (20 to 30^∘^ N, 20^∘^ W to 5^∘^ E and the spatial change in the variance across the early Holocene in CONV+VMS (*C*).

Systems that contain intrinsic abrupt thresholds should, in theory, show evidence of critical slowing as they approach a particular tipping point (e.g., ref. 3). One signature of this is an increase in variability as the system reaches a threshold (23). The variance of the vegetation fraction (using the ±500-y filtered signal) averaged over the north Western Sahara (20 to 30^∘^ N, 20^∘^ W to 5^∘^ E) increases between 9.5 and 6.5 ka BP in the +CONV+VMS simulation. The variance increase in +CONV+VMS is consistent with the abrupt behavior at around 7.5 and 6.5 ka BP and appears to signify bistability in the system. Conversely, the remaining three model configurations show constant or even decreasing variance (Fig. 4), suggesting a monostable system with no tipping points. This demonstrates that subtle parameter changes in ESMs/GCMs can strongly influence the emergent properties relevant to abrupt climate phenomena.

The spatial change in variance across the early Holocene is shown in Fig. 4*C*. The increase is strongest in the Western Sahara, where the land–atmosphere feedbacks are strong due to pronounced albedo contrasts between vegetation and the high-mineral-content soils and where precipitation is weakest today. The abrupt changes here are consistent with other paleoclimate evidence that suggests that further eastward and to the south, the reconstructed hydrological changes are generally much more gradual (20, 21).

We performed additional sensitivity simulations without interactive vegetation to evaluate the influence of vegetation cover on the precipitation response over North Africa (*Materials and Methods*). These diagnostic simulations show a strong dependence of the precipitation over North Africa (20 to 30^∘^ N, 20^∘^ W to 15^∘^ E) on the fraction of vegetation (see also *SI Appendix*, Fig. S6). The rate of precipitation increase per fractional vegetation cover is 276 mm/y for the 8 ka simulation and 217 mm/y at 6 ka after the vegetation has contracted. These values are consistent with a strong land–atmosphere coupling in this region (24). Together with the apparent threshold discussed above, this points to a dominant control by vegetation on the abrupt dynamics in the system. The reasonable agreement with the hydrogen-isotope precipitation reconstruction shown in Fig. 1 suggests that this may also be the case for the real system.

## Discussion

Valdes (2011) (7) argued that climate models are overly stable and, hence, probably unable to realistically capture abrupt events, such as those seen in the paleoclimate record. This may be because GCMs are implicitly biased toward stability through their evaluation solely against contemporary observational targets (8). While there has been some progress in simulating abrupt behavior of the Atlantic ocean circulation (54, 55), it remains unclear how model boundary conditions, parameter settings, or the span of resolved (bio-)physical processes will combine to provide a realistic representation of abrupt climate change more widely. This is therefore a critical research question, given the investment in GCM/ESM development and the potential for high-impact or catastrophic system changes in the future (4, 6).

Here, we show that a realistic and spontaneous collapse of the greening of the Sahara occurs only in the model version, which has been optimized with both present-day observations and mid-Holocene reconstructions. The successful replication of this event by the model demonstrates that coupled GCMs, like the one used here, probably resolve the required spectrum of biophysical processes. These include initiation and environmental mixing of convection, biophysical coupling to land surface, and coupling with the large-scale circulation. However, in the standard model version, these processes have potentially been misspecified, probably because present-day observations provide limited constraints for how these coupled processes operate during abrupt events (37).

This example should motivate further work with different paleoclimate events covering a wider range of systems and feedbacks. It would be beneficial to apply these developments in other ESMs to test the universality of parameterizations and to see whether this could reduce intermodel uncertainty in future projections. Different statistical approaches to model tuning can also be beneficial. Future work may consider an explicit focus on transient climate changes and on model-emulation techniques. A key development would be to robustly integrate across a hierarchy of models, from idealized atmospheric dynamical schemes (56) to lower-resolution GCMs (40) and more computationally costly ESMs, with, for example, updated cloud microphysics (57).

The absence of appreciable abrupt climate events during the instrumental era and probably several preceding centuries could be taken as evidence that such events were unlikely in the late Holocene preindustrial climate state. Future climate change will almost certainly increase the probability of encountering threshold and tipping points. Our results show the value of paleoclimate information and that present-day climate is a necessary, but insufficient, constraint on the behavior of ESMs, particularly for tipping events, where there are no parallels in the historical record. We have demonstrated that “paleoclimate tuning” of ESMs can radically improve their ability to simulate past abrupt transitions, potentially giving us more confidence in their performance for future projections.

## Materials and Methods

### Coupled GCM

In this study, we use the coupled GCM HadCM3-M2.1aD (40), which is based closely on HadCM3 (38, 58) coupled to version 2.1 of MOSES, a precursor of the current UK land surface model Joint UK Land Environment Simulator (39). The atmospheric model (HadAM3) has a horizontal resolution of 3.75× 2.5^∘^ (longitude–latitude) with 19 unequally spaced vertical levels. It uses a Eulerian hydrostatic dynamical core with schemes for convection, turbulence, gravity waves, large-scale clouds and precipitation, and cloud microphysics (40). The ocean model has a horizontal resolution of 1.25× 1.25^∘^ with 20 vertical levels. It solves the primitive equations using a rigid-lid formulation. It includes parameterizations of sea-ice leads, isopycnal eddy mixing, and the ocean mixed-layer. The atmosphere and ocean are coupled on a daily basis with no flux corrections (38).

MOSES 2.1 represents the land surface as a tiled patchwork of nine land-cover types: five PFTs: broadleaf and needle-leaf trees, C3 and C4 grasses, and shrubs; and four nonplant cover types: lakes, urban, bare ground, and ice. This model includes the dynamic vegetation scheme TRIFFID (39) so that plant coverage, structure, and productivity are coupled to the physical climate. PFT coverage is updated every 10 model days using a Lotka–Volterra competition formulation with a hierarchy of trees–shrubs–grasses (39). In this version, the vegetation respiration rate is reduced at higher temperatures following developments in MOSES 2.2. This is important in warmer climates, especially over the Amazon (40). HadCM3-M2.1 does not include dynamic dust, but this is unlikely to be critical for the north African monsoon in the mid-Holocene. Ref. 34 showed that most climate models overestimate the absorption of shortwave radiation by dust because these models rely on outdated dust optical parameters (34), leading to unrealistically large impacts on the radiation budget and, hence, precipitation.

HadCM3-M2.1aD is used here in four configurations. In addition to the standard version (STD) evaluated before (40), we introduce changes to convection (CONV), as described in ref. 37, and vegetation moisture stress (VMS), both described in more detail in *SI Appendix*. In CONV, convective entrainment/mixing detrainment is reduced at lower atmospheric levels and increased further aloft. This enhances the mass flux anomalies and thereby increases the sensitivity of monsoonal precipitation to insolation. VMS uses an updated parameterization of vegetation moisture stress, which has been optimized to reproduce the climate–vegetation coverage relationship in the tropics in comparison with satellite-observed distribution of vegetation and to allow the dynamic simulation of a “green” Sahara for mid-Holocene (6 ka BP) conditions. The fourth version combines both the convection and moisture stress changes. These model versions are labeled STD, +CONV, +VMS, and +CONV+VMS, respectively, and are summarized in Table 1.

**Table 1:** Configuration of the four GCM setups tested in this study

Run name	Orbit	GHGs	Ice and sea-level	Convection	Moisture stress	Length, y
STD	B78^*^	Ice-core	ICE-6G	Standard^†^	Standard	10,000
+CONV	B78	Ice-core	ICE-6G	CONV^‡^	Standard	10,000
+VMS	B78	Ice-core	ICE-6G	Standard	VMS^§^	10,000
+CONV+VMS	B78	Ice-core	ICE-6G	CONV	VMS	10,000

GHGs, greenhouse gases.

^*^B78: Berger (1978) (62).

^†^Standard HadCM3 mass-flux convection parameterization.

^‡^Optimized against mid-Holocene precipitation reconstructions (37).

^§^Optimized against observed present-day and reconstructed mid-Holocene tropical vegetation coverage (*SI Appendix*).

### Transient Climate Forcings

Four 10,000-y transient Holocene simulations were performed with HadCM3-M2.1. The four configurations were forced with time-dependent evolution of land-ice and sea-level derived from ICE-6G (59, 60), trace gas concentrations of CO_2_, CH_4_, and N_2_ O from ice-cores adjusted to the Antarctic Ice Core Chronology 2012 (61) and with the post-Industrialization rises replaced with the piControl mixing ratios recommended for the Coupled Model Intercomparison Project 6 and changes in the orbital parameters (62). The ice-sheet area and coastlines are updated every 500 y. Other forcings are updated every model time step. The time series of these forcings are shown in *SI Appendix*, Figs. S1 and S2. All other model boundary conditions remain constant through time at their preindustrial settings. The solar constant was updated to 1,361 Wm^– 2^. Volcanic eruptions and anthropogenic land use are not included. This setup is consistent with the PMIP4 deglaciation transient experiments described by ref. 63. The initial conditions are identical in each case and are taken from the 10 ka BP state of a PMIP4 deglaciation simulations with HadCM3-M2.1, in which freshwater is routed from Eurasian ice-sheets to the Arctic Ocean. The atmospheric state and vegetation adjust rapidly to the parameter changes, so that differences between the four simulations are visible from the first few simulated months of Fig. 4. The influence from the definition of the seasons over the past 10,000 y has been evaluated (64). This calendar adjustment applied to the JJAS time series for North Africa is smaller than 2% when a 100-y running mean is applied.

A transient cooling event lasting around 200 y is visible in all model simulations at 8 ka BP. In these transient simulations, the land–sea mask is updated every 500 y. At 8 ka BP, the Hudson Bay becomes transiently connected to the Arctic Ocean. This allows a relatively large volume of freshwater to mix into the Atlantic, causing a weakening of the Atlantic Meridional Overturning circulation (AMOC) for around 100 y. The impact on precipitation over Africa is short-lived, with an ∼60-y reduction in precipitation by around 1 mm×d^– 1^. The oscillations that precede the eventual collapse at 6 ka BP and that are associated with the biogeophysical albedo feedback are longer in duration and mostly larger in amplitude. For example, events at 7.7 ka BP, 6.7 to 6.55 ka BP, and 6.5 ka BP are larger and last between 150 and 300 y. Since the AMOC recovers within 150 y after 8 ka BP, we believe that the freshwater input is not responsible for the hydrological cycle variations evaluated here.

### Evaluating the Role of Dynamic Vegetation

We reran 150-y segments every 1,000 y of the transient simulation (+CONV+VMS) to diagnose the role of vegetation change in the precipitation response. We deactivated dynamic vegetation in three sets of simulations. The first was initialized with the simulated vegetation coverage and the second and third with the observed present-day distribution. In the third, the soil albedo over the Sahara was set equal to the clear-sky surface albedo produced by the vegetation field in the first set. The difference between the first and second configuration quantifies the vegetation feedback to first order. The difference between the second and third configurations allows a separation of the vegetation feedback into components due to vegetation-induced changes in albedo and in land-surface moisture recycling.

## Data Availability

Simulation output data have been deposited in the Bristol Research Initiative for the Dynamic Global Environment repository (https://www.paleo.bristol.ac.uk/ummodel/scripts/papers/) (65). The Met Office released the HadCM3 source code via the Ported Unified Model release (https://www.metoffice.gov.uk/research/approach/collaboration/unified-model/partnership). Code modifications required to produce the standard version (here, STD) are available from Geoscientific Model Development, https://doi.org/10.5194/gmd-10-3715-2017. The code changes and parameter namelist files required for CONV are available from Figshare, https://dx.doi.org/10.6084/m9.figshare.12311360 and are labeled REVopt. The code changes and parameter namelist files required for VMS are available from Figshare, https://doi.org/10.6084/m9.figshare.13650062.v1.
